# With or without us? An audit of disability research in the southern African region

**DOI:** 10.4102/ajod.v3i2.76

**Published:** 2014-06-04

**Authors:** Judith McKenzie, Gubela Mji, Siphokazi Gcaza

**Affiliations:** 1 Centre for Rehabilitation Studies, Stellenbosch University, South Africa; 2 Disability Studies Programme, University of Cape Town, South Africa

## Abstract

**Background:**

Disability research in the global South has not received significant critical consideration as to how it can be used to challenge the oppression and marginalisation of people with disabilities in low-income and middle-income countries. The Southern Africa Federation of the Disabled (SAFOD) embarked on a programme to use research to influence policy and practice relating to people with disabilities in Southern Africa, and commissioned an audit on research expertise in the region. In this article, a research audit is reported on and situated in a framework of emancipatory research.

**Objectives:**

This article sets out to describe a preliminary audit of disability research in the southern African region and to draw conclusions about the current state of disability research in the region and make recommendations.

**Method:**

The research method entailed working with disability researchers in the ten SAFOD member countries and utilising African disability networks hosted on electronic media. Disability researchers working in the region completed 87 questionnaires, which were reviewed through a thematic analysis.

**Results:**

The discussion of results provides a consideration of definitions of disability; the understanding of disability rights, research topics and methodologies; the participation of people with disabilities in research; and the challenges and opportunities for using research to inform disability activism.

**Conclusion:**

The conclusion highlights critical issues for future research in the region, and considers how a disability researcher database can be used as a tool for disability organisations to prioritise research that serves a disability rights agenda.

## Introduction

The United Nations Convention on the Rights of Persons with Disabilities (UNCRPD), which has now been entered into international law, is a significant step towards realising the rights of people with disabilities (United Nations [UN] 2006). The Convention seeks to address discrimination, change perceptions and combat stereotypes and prejudices. It also places an obligation on governments to ensure that they assist people with disabilities to achieve a state of equality with the citizenry without disabilities of each of their countries. Article 31 of the Convention notes the importance of states gathering research data that can inform policy and monitor progress towards the realisation of the rights of people with disabilities. People with disabilities need to be able to monitor and evaluate the impact of UNCRPD on their lives, and involvement in research will give them the impetus to do so.

This article reports on a research audit commissioned by the Southern Africa Federation of the Disabled (SAFOD), an umbrella human rights organisation for people with disabilities, for the Centre for Rehabilitation Studies (CRS) at the University of Stellenbosch to conduct. We begin with an analysis of the emancipatory research perspective of SAFOD, before presenting the study and our conclusion and recommendations.

## Emancipatory research

In 2006 SAFOD initiated the SAFOD Research Programme (SRP) with the following aims:

- to engage in partnerships with researchers in community-based and academic research in a spirit of co-operation and trust
- to develop the capacity of people with disabilities as research partners
- to use research to develop effective pro-poor policy and practice affecting people with disabilities in the region.

The SRP is concerned with the relationship between people with disabilities and researchers, with the aim that people with disabilities become partners in research that has a practical application in their lives. For this reason, the research audit was conducted within the framework of an emancipatory paradigm. Emancipatory research (1) is based on a social model of disability, (2) engages people with disabilities in all stages of the process, and (3) has a transformative aim (Barnes 2008). Each of these issues will be discussed below with regard to the disability research audit.

### Social model of disability

The *World report on disability* (World Health Organization [WHO] 2011) outlines the development of a social model of disability, largely through the efforts of people with disabilities themselves, in reaction to the medicalisation of disability by health professionals. Different ‘models’ of disability have been positioned as being dichotomous, thereby stifling dialogue about the relative impact of impairment and environment (Grue 2011). The International Classification of Functioning, Disability and Health (ICF) is presented in the *World report on disability* as a framework that can begin to bridge this gap, recognising both the nature of impairment and the importance of environmental factors. The environmental factors within the life-situation of a person with an impairment may either pose barriers or be a facilitator to participation (WHO 2002). Within the UNCRPD, such barriers are seen as a form of social injustice that states have an obligation to address (UN 2006). For Barnes (2008), an emancipatory research approach must at least include a focus on environmental barriers, and must prioritise the knowledge and experience of people with disabilities.

### The engagement of people with disabilities

The involvement of people with disabilities in disability research can be seen as existing along a continuum, ranging from weak to strong engagement. At the weak end of the scale, involvement would be in the form of researchers engaging with people with disabilities merely as subjects of research or perhaps for consultation at an advanced stage of research. At the strong end of the scale, disabled people’s organisations (DPOs) would be involved in setting the research agenda as well as in conducting, commissioning and disseminating the research. This was found to be true in a specific European context; amongst people with disabilities there was ‘in particular, a desire to be involved in shaping research agendas and defining research questions whilst valuing the methodological expertise and credibility of academic researchers’ (Priestley, Waddington & Bessozi 2010:741). The ownership of research is a contested area. DPOs have often complained that academic researchers use the products of their research for career advancement rather than for the emancipation of people with disabilities (Garbutt & Seymour 1998).

### Transformative aim

Carmichael (2004) points out that research is a means to an end, not an end in itself. Research must be communicated in such a way that it provides evidence to action, as opposed to being relegated to a dusty shelf. Chalklen, Seutloadi and Sadek (2009) found that in the southern African context, disability research is not sufficiently solution focused and does not provide material for advocacy because it is pitched at a too generic level. According to these authors, statistics are not disaggregated in such a way as to make them usable for disability activists. These criticisms must be addressed if researchers are to contribute to the empowerment of people with disabilities in the manner envisaged in Article 31 of the UNCRPD.

It is within this framework that the African Network for Evidence to Action in Disability (AfriNEAD) has been developed. This network seeks to bridge the gap across a broad range of issues relevant to realising the rights of people with disabilities (Mji *et al*. 2009). One of the key reasons for the development of AfriNEAD was to investigate the quality and the suitability of existing disability research. According to AfriNEAD, the challenge is clear: it is not just more research that is needed; it is ‘improved’ research and research that can be translated into policy and practice. Translating research into evidence-based advocacy, policy, practice and products – particularly in the pan-African context – needs to be systematically addressed in a co-ordinated, coherent and consistent fashion. It is only when this happens that research evidence can act as a springboard for human rights instruments such as the UNCRPD (Mji *et al*. 2009).

## Research method

### Data collection

A questionnaire, summarised in Table 1, was developed within the framework of an emancipatory research paradigm.

**TABLE 1:** Summary of questionnaire.

Section	Items
Demographics	Name, Telephone, Fax, Cell number, Email address, Physical address (including country), Employing university/organisation, Faculty/department/section, Position held in the organisation
Understanding of disability	What definition of disability are you working with in your research? What are the environments in which your research is carried out? For example, rural communities, hospital, clinical trials, et cetera. What do you understand about emancipatory disability research?
Engagement with people with disabilities	Are people with disabilities involved in your research? In what role or capacity?
Transformative nature of research	How do you disseminate your research findings? What formats and forums do you use? Which audiences do you target? In what ways has your research been useful in realising the rights of people with disabilities? What do you see as the challenges facing researchers in using research evidence to realise the rights of people with disabilities? What suggestions do you have to address these challenges? In what ways does your research relate to government policy development? What do you see as current critical issues in disability research?
Conducting research	What research methodologies do you use? What topics have you researched? What procedures do you follow to get ethical approval for your research? What international partnerships have you engaged in?

Initially, the questionnaire was sent electronically to a group of researchers in the region who were well-known to SAFOD, and who had participated or advised in the development of the SRP. In addition, we engaged with a research capacity-building programme run by the Department of Psychology at the University of Stellenbosch and SAFOD. The programme was run over two years on a block-release system with the aim of:

… build[*ing*] the institutional capacity of the organisation (SAFOD) to design, drive and deliver their own research and development programme, focusing on disability issues with an inclusive poverty, emancipation, social exclusion and human rights focus. (University of Stellenbosch 2007)

Trainees were people with disabilities recruited by DPOs in the SAFOD member countries. SRP trainees gave their inputs to the questionnaire’s development and agreed to approach at least five researchers in their own countries to complete the questionnaire; they were paid for their work. They administered questionnaires electronically or in face-to-face interviews, depending on the availability of the respondents. All respondents provided contact details of other suitable respondents to create a snowball sampling effect. Whilst some of these researchers were connected to SAFOD, others were not. The respondents gave informed consent to their participation in the research audit. This process yielded a total of 87 questionnaires.

### Data analysis

A database was created to store researchers’ names, contact information, research methodologies and the topics of their research. Data obtained from the questionnaire was grouped into response categories derived from the emancipatory paradigm (understanding of disability, engagement with people with disabilities and transformative aim). Each researcher analysed a response category and then validated their findings with the other two researchers.

The response categories were analysed as follows:

- Identifying critical or emerging themes in each category.
- Grouping together statements that supported the identified theme.
- Identifying the frequency of statements in each theme (counting the number of responses and converting it into percentages). The rationale for identifying the frequency of statements was to indicate the dominant trends within this very specific sample of respondents. Whilst we make no claim as to the generalisability of these trends, we find it worthwhile commenting on their occurrence and considering future directions in the light of these findings.

## Results

The researchers came from throughout the region; the highest number came from South Africa, followed by Zimbabwe and Botswana, and the lowest number came from Angola and Namibia (see Table 2).

**TABLE 2:** Country of residence of researchers.

Country	Number of respondents
South Africa	16
Zimbabwe	14
Botswana	11
Zambia	10
Lesotho	9
Mozambique	8
Malawi	5
Angola	4
Swaziland	4
Namibia	3
Non-resident in Africa	3

**Total**	**87**

Most researchers (46%) were employed by universities or colleges (though not necessarily full-time). Government was the next most common employer (14%), followed by non-governmental organisations (11%), and DPOs and private consultancies (10% each). The remaining 9% were based in national research institutes.

### Definitions or models of disability

Evidence from respondents (as illustrated in Table 3) indicates that the definition of ‘disability’ is still a contested and complex issue. Respondents had different understandings and orientations, at times moving between definitions.

**TABLE 3:** Definitions of disability.

Definition of disability	Number of responses within theme	%
Medical model definition	23	35
Social model definition	20	31
ICF definition	12	18
UNCRPD definition	5	8
Non-specific	5	8

**Total**	**65**	**100**

ICF, International Classification of Functioning; UNCRPD, United Nations Convention on the Rights of Persons with Disabilities.

The medical model was the most common definition used by researchers, but only marginally more common than the social model. Many researchers adopted the ICF definition, which incorporates elements of the medical and social models: ‘This is a complex question and I can’t answer it generically, but let’s say social model and ICF’ (Respondent 5, male, academic).

### Involvement of people with disabilities

The majority of respondents involved people with disabilities in some aspect of the research process, albeit at different levels and in varying roles and capacities (see Table 4).

**TABLE 4:** Involvement of people with disabilities in research.

Different levels of involvement of people with disabilities	Number of responses within theme	%
Participants/respondents/interviewees	25	31
Research assistants/data collectors/enumerators/tutors	18	23
Partnerships/collaborators/commissioning agencies/reference group members	10	12
Researchers/investigators/co-researchers/consultants/senior research staff/senior academic staff	10	12
Involvement in the research process/full involvement (design, implementation, analysis and dissemination of findings)	7	9
Organisational	6	7
No involvement/no involvement yet	5	6

**Total**	**81**	**100**

### Promotion of human rights

There is a strong indication that researchers are attempting to promote the human rights of people with disabilities, as indicated in Table 5.

**TABLE 5:** The understanding of human rights.

Contribution of research to rights of people with disabilities	Number of responses	%
Improving service delivery	17	19
Contributing to the development of public policy and mainstreaming in global development initiatives	15	17
Not sure	8	9
Identifying best practice and evidence to action	7	8
Focusing on specific sectors to promote inclusion (education, employment, rehabilitation)	7	8
Empowering people with disabilities and giving them a voice	7	8
Expanding awareness of disability as a social construct and as a human rights issue	6	7
Awareness and monitoring of UNCRPD	5	6
Examining conditions of living	5	6
Developing training manuals for dealing with disability issues	4	4
Highlighting issues of sexuality and HIV/AIDS	4	4
Promoting social inclusion	4	4

**Total**	**89**	**100**

UNCRPD, United Nations Convention on the Rights of Persons with Disabilities.

### Challenges of using research evidence to action

Most respondents reported that there is limited capacity (both human and financial) for service providers to conduct research (see Table 6).

**TABLE 6:** Challenges of using research evidence to action.

Categories showing respondents’ perceptions regarding challenges of using research evidence	Number of responses within theme	%
Problems with quality of research evidence	39	31
Problems related to funding, access of people with disabilities, communication, time and material, and resources to translate research evidence into practice	34	27
Politician/policy-maker attitudes, knowledge and lack of political will	30	24
Lack of participation of disabled people’s organisation	12	10
Conflicting opinions to no opinions	11	9

**Total**	**126**	**100**

### Suggestions for using research evidence

Some respondents felt that there should be a clear definition and delineation of roles between researchers and activists. In this regard, researchers have as their main objective the generation of new knowledge, whilst DPOs are advocates for their constituencies (see Table 7).

**TABLE 7:** Suggestions for using research evidence.

Suggestions for using research evidence	Number of responses	%
Development of meaningful partnerships between DPOs and researchers	29	27
Development of a dissemination and utilisation strategy	23	21
Education and training of DPOs	16	15
Mainstreaming disability in all sectors	16	15
Involvement of policy-makers in the disability research agenda	15	14
No response or not sure how to respond	6	5
Clear definition of roles	3	3

**Total**	**108**	**100**

DPOs, disabled people’s organisations.

### Policy development

Respondents saw a strong connection between evidence to action and influencing government policy (see Table 8).

**TABLE 8:** Respondents involved in government policy development.

Respondents’ perceptions of their involvement in and influence on government policy	Number of responses	%
Described their involvement in and influence on government policy	52	57
Described lack of involvement and frustration at lack of involvement in government policies	14	16
Identified gaps between research evidence and policy development	10	11
Other	13	15

**Total**	**89**	**100**

## Discussion

It appears that an interactional, human rights understanding of disability is gaining wider acceptance in the region. The UNCRPD and the ICF are the most prominent instruments underpinning disability research. Although there are important differences between these two approaches that will not be discussed here, both are shifting the focus of research to a greater exploration of the environment in which disability occurs or is created. A more impairment-oriented, medical approach to disability is evident in countries that do not have access to the wide range of literature available in the English language (e.g. Mozambique and Angola).

There was a call, specifically regarding the ICF, to adopt a working definition of disability, so as to enable researchers to design studies that are comparable internationally; and in so doing, describe and monitor the implementation of the UNCRPD. However, other researchers draw more directly on a framework of social justice and equalisation of opportunity. In addition, some researchers make a plea for recognising indigenous knowledge and African perspectives in disability research. It appears that it would be premature to end the debate at this point.

Amongst respondents there was a strong recognition of the need to involve people with disabilities at all levels of the research process. Some were concerned about the call for full participation of people with disabilities in research without stipulating their role in research. In the African context, not all people with disabilities have had formal education, and are at a disadvantage with regard to technical research skills. Whilst the development of these skills might take place for those people with disabilities expressly interested in their development, it was proposed that the aim for the disability movement overall should be to increase the capacity of people with disabilities to engage with researchers, to utilise research and to ask the right questions. Furthermore, a deeper knowledge of the African context and indigenous knowledge systems could be integrated into the research process (Owusu-Ansah & Mji 2013).

An evidence to action approach is required to ensure that research benefits and makes a difference in the lives of people with disabilities. Dissemination of research findings must be targeted to reach the intended people, building knowledge of the rights and responsibilities of people with disabilities (Barnes 2008; UN 2006). Where there was limited disability research capacity (e.g. in Namibia) it was noted that there was research in existence that could be relevant to supporting equal opportunities for people with disabilities, if the data were disaggregated for disability. Thus, disability research should be undertaken with appropriate strategies and funding for dissemination and advocacy from the start, rather than seeing these as add-on, optional activities to be performed at the end of the research project.

The process of translation of research evidence raises questions regarding the origin of research questions. Lately, disability research participants have become interested in knowing from researchers how their research outcomes will be used to address the needs and priorities of people with disabilities. Though a daunting prospect, this opens a new and exciting space for the inclusion of people with disabilities in the critical planning of research for better accountability and impact (Priestley *et al*. 2010).

At the core of these arguments is the need for equalisation of opportunities for people with disabilities. We believe that the discourse regarding research evidence should not be a fixed entity, but rather a fluid construct that is subject to the context and changing theoretical and socio-political understandings of disability in that area (Owusu-Ansah & Mji 2013). We contend that it is not the methodology that is used that determines the effectiveness of research in transforming the lives of people with disabilities; rather, it is the consciousness of the central place of the struggles of people with disabilities and their families to realise the rights that have been outlined in the UNCRPD.

## References

[CIT0001] Barnes C, 2008, ‘An ethical agenda in disability research: Rhetoric or reality?’, in Mertens D.M. & Ginsberg P.E. (eds.), *The handbook of social research ethics*, pp. 458–473, Sage, London.

[CIT0002] Carmichael A, 2004, ‘The social model, the emancipatory paradigm and user involvement’, in Barnes C. & Mercer G. (eds.), *Implementing the social model of disability: Theory and research*, pp. 191–207, The Disability Press, Leeds.

[CIT0003] Chalklen S., Seutloadi K. & Sadek S, 2009, *Literature review*, Southern African Federation of the Disabled, Bulawayo.

[CIT0004] Garbutt R. & Seymour J, 1998, ‘“Do we all get a PhD?” Attempting emancipatory research relating to disability in an academic environment’, paper presented at the British Sociological Association Conference, Edinburgh, viewed 05 July 2013, from http://disability-studies.leeds.ac.uk/files/library/garbutt-Do-we-all-get-a-PhD-2-.pdf

[CIT0005] Grue J, 2011, ‘Discourse analysis and disability: Some topics and issues’, *Discourse & Society* 22, 532–546. http://dx.doi.org/10.1177/0957926511405572

[CIT0006] Mji G., MacLachlan M., Melling-Williams N. & Gcaza S, 2009, ‘Realising the rights of disabled people in Africa: An introduction to the special issue’, *Disability & Rehabilitation* 31(1), 1–6. http://dx.doi.org/10.1080/09638280802280288 19194806 10.1080/09638280802280288

[CIT0007] Owusu-Ansah F.E. & Mji G, 2013, ‘African indigenous knowledge and research’, *African Journal of Disability* 2, 5 pages. http://dx.doi.org/10.4102/ajod.v2i1.30 10.4102/ajod.v2i1.30 PMC5442578 28729984

[CIT0008] Priestley M., Waddington L. & Bessozi C, 2010, ‘Towards an agenda for disability research in Europe: Learning from disabled people’s organisations’, *Disability & Society* 25, 731–746. http://dx.doi.org/10.1080/09687599.2010.505749

[CIT0009] United Nations (UN), 2006, *Convention on the rights of persons with disabilities*, United Nations, New York.

[CIT0010] University of Stellenbosch, 2007, *Department of Psychology: Community projects*, viewed 08 July 2012, from http://sun025.sun.ac.za/portal/page/portal/Arts/Departments/psychology/community

[CIT0011] World Health Organization (WHO), 2002, *Towards a common language for functioning, disability and health ICF*, World Health Organization, Geneva.

[CIT0012] World Health Organization (WHO), 2011, *World report on disability*, World Health Organization & World Bank, Geneva.

